# Case Report: Unique management strategy for rare case of esophageal foreign body

**DOI:** 10.3389/fsurg.2024.1370876

**Published:** 2024-03-05

**Authors:** Dastan Rustemov, Ruslan Bilal, Ruslan Tukinov, Adilzhan Nekessov, Damir Dzhenalaev, Erbulat Ermeshev, Zarip Mukhamedov, Dulat Mustafinov, Ruslan Utebaliev, Zhenis Sakuov, Baurzhan Kaliev

**Affiliations:** ^1^ Clinical Academic Department of Pediatric Surgery, National Research Center for Maternal and Child Health, University Medical Center, Astana, Kazakhstan; ^2^ Department of Medicine, School of Medicine, Nazarbayev University, Astana, Kazakhstan; ^3^ Clinical Academic Department of Radiology and Nuclear Medicine, National Research Center for Maternal and Child Health, University Medical Center, Astana, Kazakhstan; ^4^ Resuscitation and Intensive Care Unit, Clinical Academic Department Pediatric Anesthesiology, National Research Center for Maternal and Child Health, University Medical Center, Astana, Kazakhstan

**Keywords:** foreign body, post-traumatic aneurysm of aorta, esophageal perforation, endoscopy, Case Report

## Abstract

**Background:**

Foreign bodies that enter the esophagus can cause serious complications that may require extensive surgical intervention, including open surgery. The treatment method depends on the location, size, configuration, and number of foreign bodies in the esophagus, but to date, the best method to remove foreign bodies from the esophagus remains uncertain. Foreign bodies which can damage the walls of esophagus varies from bones and bone fragments, to metallic objects and batteries. In this article, we present a rare case of a “fish bone” penetrating through the esophagus walls and directly punctured the aorta, forming a post-traumatic saccular pseudoaneurysm of the descending thoracic aorta, which was successfully treated with endovascular stent placement to the aorta and with endoscopic foreign body removal.

**Case summary:**

We reported a case of a 16-year-old male with a 6-day history of chest pain after consuming fish. As result of immediate test in regional hospital using oral flexible esophagogastroduodenoscopy abnormalities were not reported. Due to persistence of beforementioned symptoms, a fiberoptic esophagogastroduodenoscopy was performed 3 days later, revealing a 1.5–2.0 cm long altered area with contact bleeding 33.0 cm from the incisors, but no visualization of any foreign body. Computed tomography revealed a fish bone that had completely passed through walls of the esophagus and punctured the aortic wall, forming an aneurysm. Patient was urgently hospitalized by air ambulance to our hospital for high-specialized medical intervention after 6 days. After the endovascular placement of a stent graft, the fish bone was successfully removed by endoscopic intervention. 12 months follow up showed no abnormalities.

**Conclusion:**

Endoscopic removal of foreign bodies in the esophagus and extracting a foreign body after thoracic endovascular aortic stent may be a feasible option for some cases.

## Introduction

Foreign bodies can accidentally enter the esophagus during food intake or accidentally swallowing of small objects by children. In the US, ingestion of foreign bodies is a common emergency condition with annual report of over 100, 000 cases (1). Due to the nature of food products in China, common foreign bodies lodged in the esophagus are fish and bird bones (2). The prevalence of foreign bodies in the esophagus among children is most common from six months to three years of age (3). A rare dangerous complication of esophageal foreign bodies is aorta-esophageal injury, which requires extensive surgical intervention (4–6). The optimal treatment for this condition is unknown as this injury is extremely rare, with few published reports on esophageal foreign bodies with aortic injury and aneurysm formation (7–14), and many patients may not receive timely medical intervention.

In this article, we describe the case of a 16-year-old child who had a fish bone that had completely passed through all layers of the esophagus and pierced the wall of the aorta, forming a post-traumatic saccular pseudoaneurysm in the descending thoracic aorta. In the emergency room oral flexible esophagogastroduodenoscopy showed changed area 1.5–2.0 cm long as well as swelling, plaque fibrin After endovascular stent-graft was placed in the thoracic aorta the fish bone was successfully removed endoscopically.

## Case presentation

### Chief complaints

A 16-year-old male child was admitted to our hospital, the University Medical Center of the National Scientific Center of Maternal and Childhood, after 7 days ingestion of the foreign body, the child was complaining of retrosternal pain radiating to the left side when eating and changing body positions, with a fever as high as 37.8°C. As reported by the parents, the boy had eaten fish, after which time pain manifested itself behind the sternum.

### History of present illness

At the time of admission to the hospital, the patient had no fever, nor any difficulty in swallowing, no bloody vomiting, no rectal bleeding, or other related symptoms.

### History of past illness

The child fell in the range of normal height and weight for the child's age and sex. There were no prior medical records found for the child and no indications in the family medical history suggesting hereditary or any other health conditions that would explain the complaints.

### Physical examination

**Figure 1:**
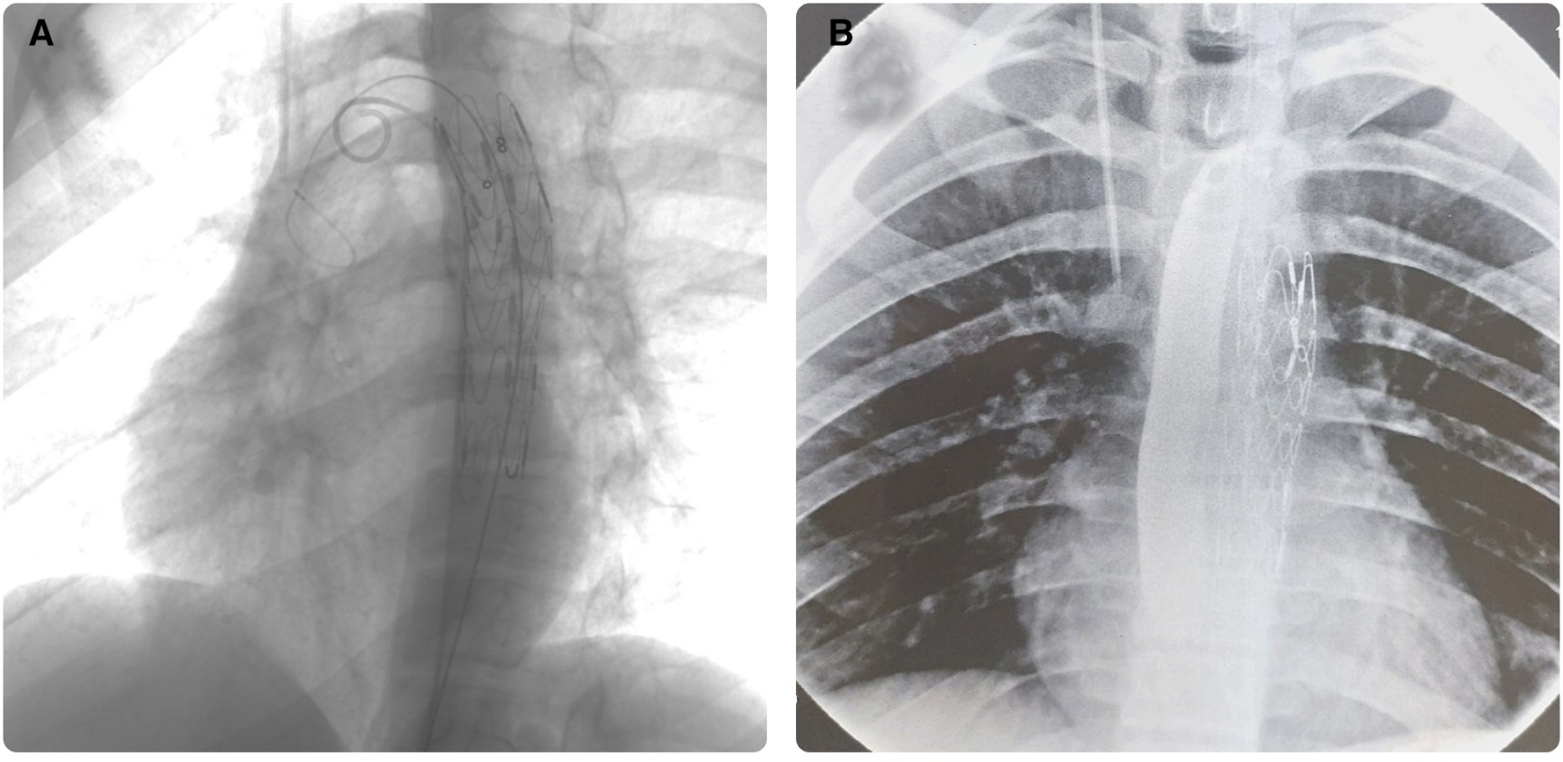
An image of the endovascular reconstruction (**A**) the image of a contrast study of the esophagus of the thoracic aorta by stent-graft placement (**B**).

The patient was admitted to the General Pediatric Surgery Department in the University Medical Center of the National Scientific Center of Maternal and Childhood. During the physical examination conducted on the child noabnormalities were detected. The child laid on his right side that alleviated the pain. Day after the hospitalization, an endovascular implantation of a stent-graft into the thoracic aorta was performed. Using a diagnostic guide wire, a diagnostic catheter Pig Tail 5 Fr was inserted into the ascending aorta, and a control aortography was performed, which showed the location of the aneurysmal dilation. A linear module of the endoprosthesis TAA2222B080 (Figure 1A) was delivered, positioned, and deployed into the aortic lumen via a stiff guide wire inserted through the right common femoral artery by arteriotomy. It was adequately positioned, confirmed during the control aortography, and the aneurysmal dilation was no longer visible. The Pig Tail 5Fr catheter and the introducer were removed.

### Laboratory examinations

Blood test results showed the following inflammatory changes: white blood cells—15.77 * 10^9 ^/L (normal range 4.50 * 10^9 ^/L–13.00 * 10^9 ^/L), C-reactive protein—101.74 mg/L (normal range 0.00 mg/L–5.00 mg/L).

### Imaging examinations

**Figure 2:**
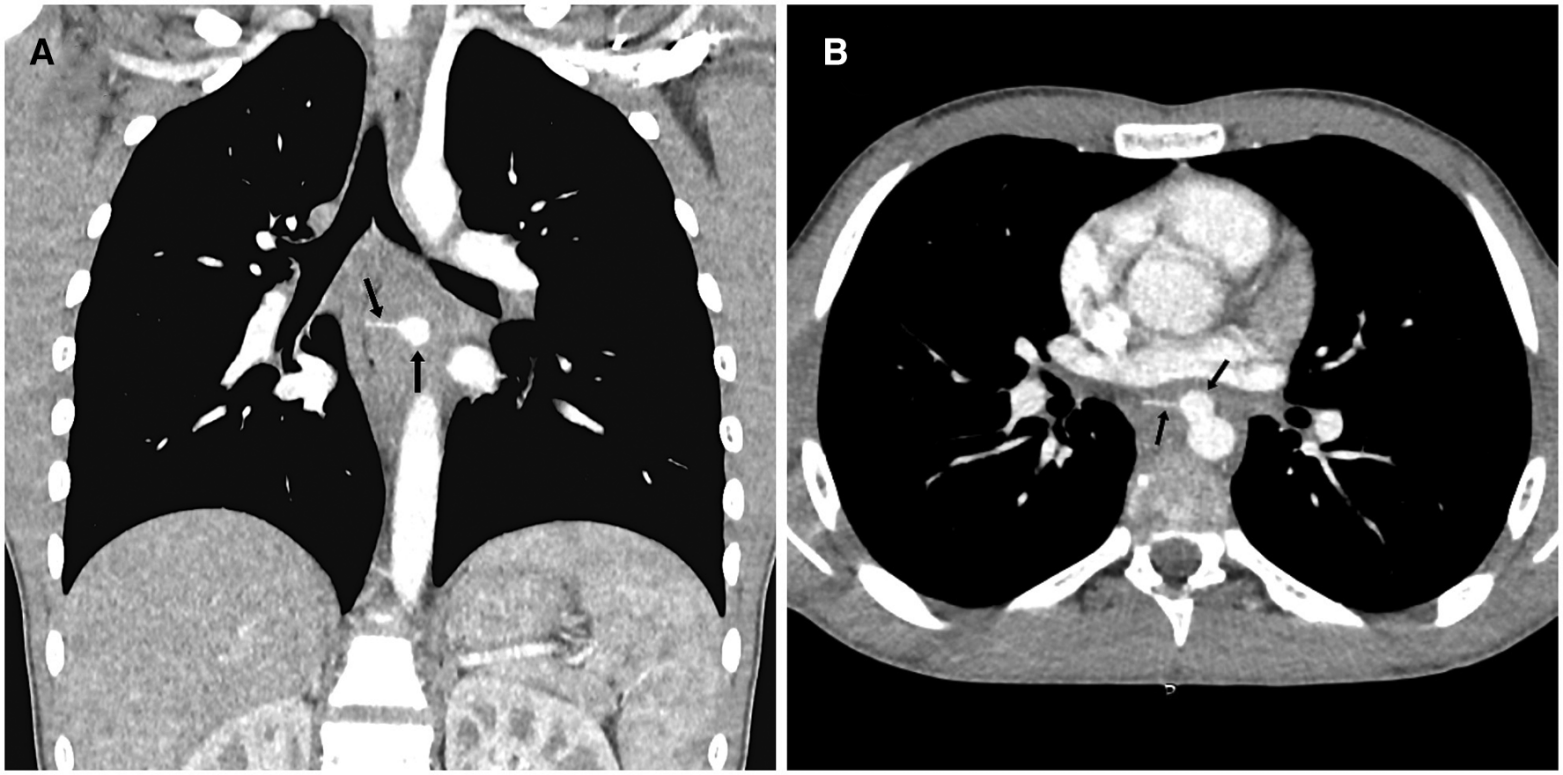
Computed tomography image with contrast.

Results of contrast-enhanced computed tomography performed on admission day showed a foreign body in the middle section of the mediastinum in the form of a fish bone (Figure 2A), a perforation of the wall of the descending thoracic aorta by a foreign body, and a formation of a post-traumatic saccular pseudoaneurysm of the descending thoracic aorta (Figure 2B).

At the level of the middle mediastinum, below the bifurcation of the bronchi, at a distance ranging from 3.6 cm and 1.67 cm from the ventral part of the Th6-Th7 intervertebral space, a transversely oriented foreign body was visible, with dimensions of 2.3 × 0.17 cm, with clear, smooth contours and a density of 184–247 Hounsfield units.

**Figure 3:**
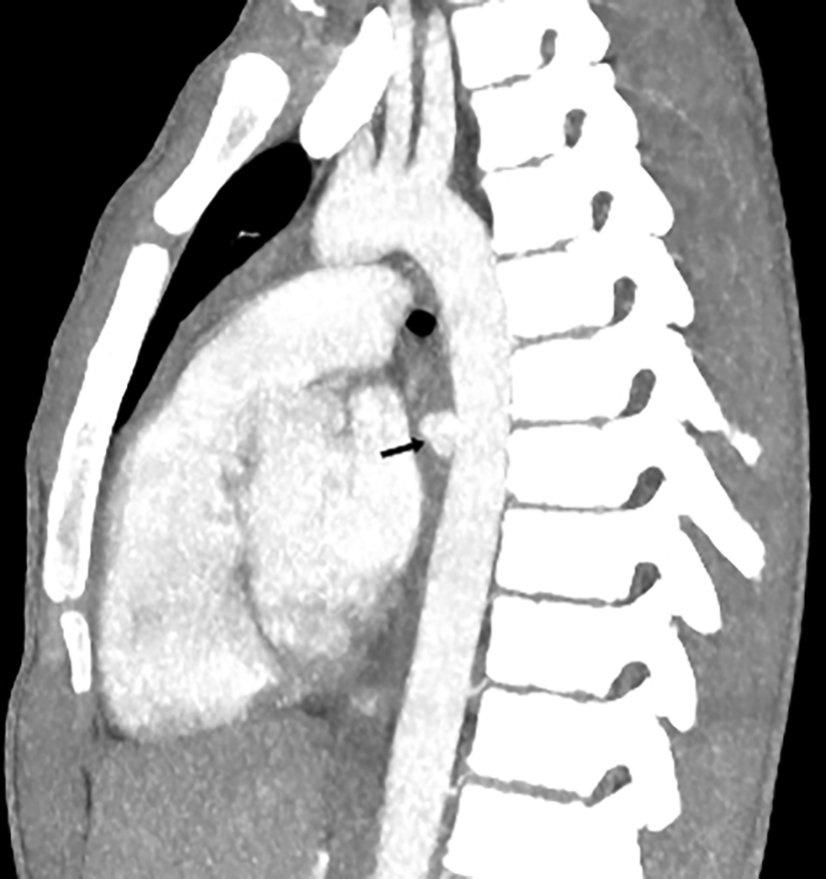
Image of contrast-enhanced computed tomography.

After the bolus intravenous contrast was administered, images were taken of the heart chambers and the major mediastinal vessels (Figure 3). At the level of the ventral portion of the descending aorta, in the projection of the Th6-Th7 intervertebral space, a saccular aneurysm with clear smooth contours measuring 1.25 × 1.06 × 1.18 cm was visible. One-third of the above-mentioned foreign body was located within the aneurysm structure. The left side end of the foreign body projected into the esophageal wall.

### Final diagnosis

A foreign body, a fish bone, was lodged in the mediastinum at the Th6-Th7 level. A false aneurysm of the thoracic aorta was caused by the fish bone in the esophagus.

### Treatment

**Figure 4:**
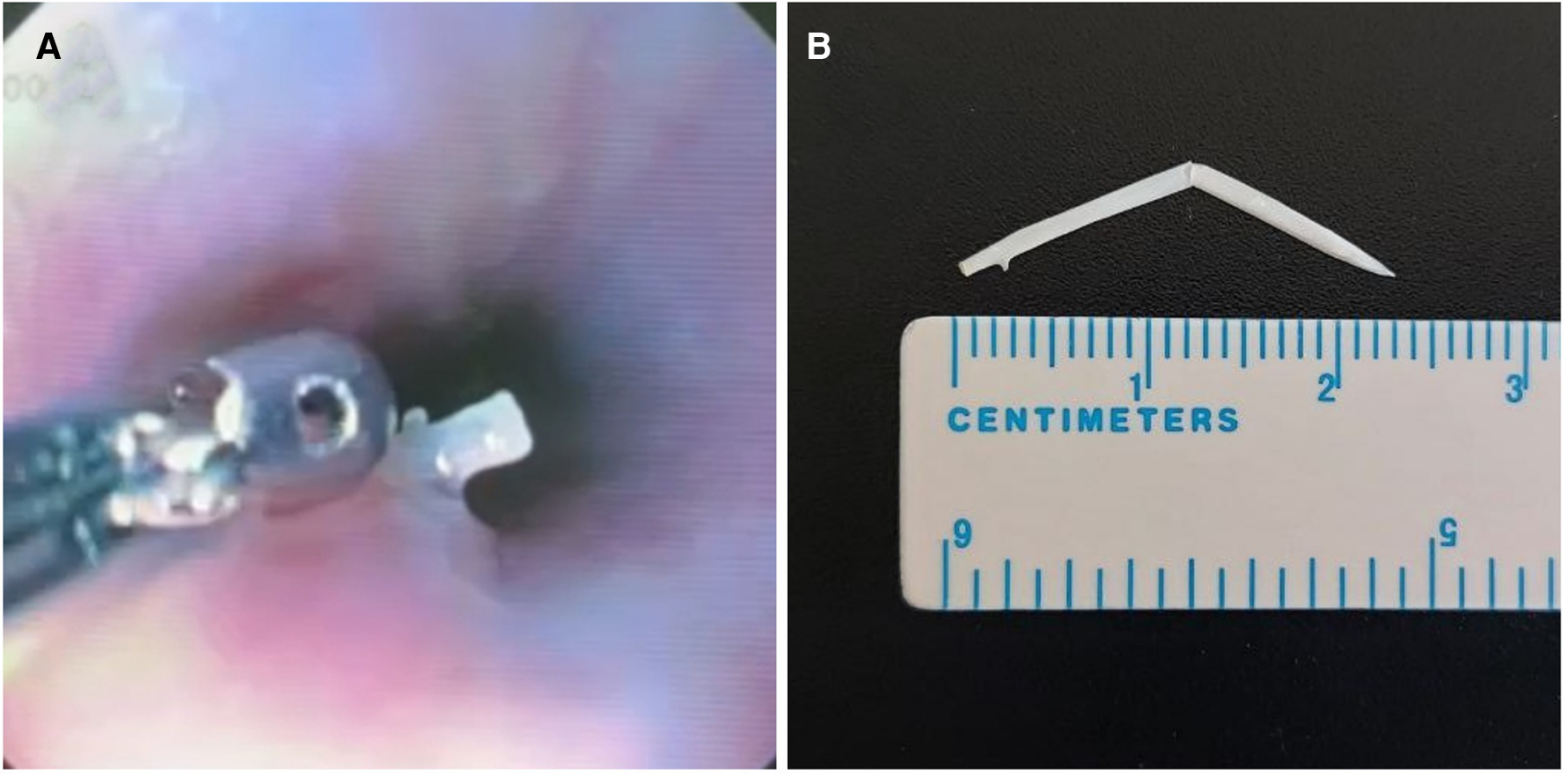
Picture of foreign body in the esophagus (fish bone).

8 day after the ingestion of foreign body, we successfully performed an endovascular implantation of a stent-graft in the thoracic aorta. Then, endoscopic examination was performed after 4 days. The tip of a white foreign hard body, the fish bone, measuring 1.7 cm × 0.3 cm, with a fixed position and smooth surface, was detected in the lumen of the esophagus, 35 cm from the anterior midline incisors. The mucous membrane around the foreign body was edematous, moderately elevated above the level of the mucosa. The foreign body was removed with biopsy forceps without technical difficulties, and the puncture site did not bleed on inspection (Figure 4A).

The length of the fish bone was approximately 25 mm (Figure 4B) and there were no fractures. There was no bleeding or pus at the site during removal. In the postoperative period, the patient continued to receive antibacterial therapy.

The contrast x-ray examination of the esophagus was performed day after the foreign body removal. Results showed that the passage of the esophagus was preserved, and no contrast leakage was detected. Figure 1B shows the condition after the stenting of the descending aorta. During contrast imaging, the esophagus showed clear and smooth contours, with preserved patency and no signs of contrast leakage. A shadow of the subclavian catheter is seen to the right, projected onto the superior vena cava.

Foreign body in the mediastinum was not differentiated from images of computed tomography after removal. A stent-graft is visible in the descending aorta at the level of vertebrae Th4-Th7. After intravenous bolus contrast administration, the heart chambers and large vessels of the mediastinum were filled with contrast and the aorta was homogeneously filled with contrast material. Signs of aneurysm are not differentiated.

### Outcome and follow-up

Currently, it has been 12 months since the surgery. The child’s condition is satisfactory.

## Conclusion

In conclusion, the formation of post-traumatic aneurysm due to foreign body ingestion can cause bleeding during the presence and removal of a foreign body. Our experience has shown that endovascular thoracic aorta stent-graft repair reduces the risk of bleeding to a minimum and strengthens the aortic wall with elaneurysm elimination. The optimal time of foreign body removal remains uncertain, depending on the factors such as location and medical complications.

## Discussion

Foreign bodies in the esophagus typically traverse the gastrointestinal tract without complications. However, accidental ingestion of fish bones represents a common occurrence in emergency departments and can give rise to diverse complications (15). If the bone damages the esophageal wall and perforates it, it can also affect adjacent structures, potentially leading to complications such as bleeding, the formation of aorto-esophageal or tracheo-esophageal fistulas, mediastinitis, abscesses, and other related issues (16, 17).

There are other causes of esophageal perforation. Most perforations occurred during instrumentation of the oesophagus (47.6%): balloon dilation-56%, endoscopic examination of the oesophagus with or without biopsy-26%, removal of foreign bodies from the oesophagus with a rigid endoscope-10%, nasogastric tubes-8%. Other causes were corrosion and trauma (17.1%). The mortality rate was 21% (18).

This is the first clinically described case of the extraction of the swallowed fish bone, which resulted in esophageal penetration and subsequent formation of a thoracic aortic aneurysm.

Fish bones can typically be removed through endoscopy, although in certain cases, extensive surgical intervention might be necessary (19). Prolonged presence of foreign bodies in the esophagus correlates with increased adverse outcomes and a higher incidence of complications. For instance, in cases of aorto-esophageal fistula, the fatality rate ranges from 40% to 60% (20). However, there are exceptions to this trend. For example, Sheung-Fat Ko and colleagues reported a case involving a 54-year-old man with a similar diagnosis to our case study, yet the patient remained asymptomatic and declined surgery (21). Six years later, a repeat computed tomography scan revealed a “fish bone” compressed within the aorta, but the patient remained in good health. Another case (7) documented acute aortic rupture with aneurysm formation complicated by mediastinitis, also caused by a fish bone. This case was successfully managed with the placement of a stent graft and thoracotomy. With the advancement of new technologies, minimally invasive interventions have become increasingly prevalent. Ruan and colleagues demonstrated successful endoscopic removal of a foreign body from the esophagus following the placement of an endovascular stent graft (22). They suggested that stent graft placement minimizes the risk of bleeding and associated complications.

Our case presented a unique scenario where a fish bone, initially lodged in the esophagus, resulted in damage to the aorta, leading to the development of a post-traumatic saccular pseudoaneurysm of the descending thoracic aorta. This pseudoaneurysm posed an elevated risk of arterial bleeding and rendered extraction challenging through standard endoscopic methods. Despite our inability to visually detect the fish bone during the initial endoscopy, we prepared for an open operation, including the possibility of a thoracotomy, with vascular and cardiac surgeons on standby.

Upon subsequent diagnostic endoscopy, the tip of the white-colored foreign body—the fish bone—became visible approximately 35 cm from the anterior midline incisors. The mucous membrane surrounding the foreign body appeared edematous and moderately elevated above the mucosa. With the aid of ultrasound guidance and endoscopic biopsy forceps, the foreign body was successfully removed without encountering technical difficulties. Importantly, no bleeding was observed in the area of the foreign body puncture upon inspection.

We hypothesize that localized inflammation of the esophageal mucosa may have induced edema, rendering the tip of the foreign body invisible during the initial endoscopy. It is conceivable that following the placement of the stent graft in the aorta, the stent might have displaced the end of the fish bone, causing it to rebound towards the esophageal wall.

The absence of clinical signs such as hyperthermia and intoxication, coupled with the lack of evidence of mediastinitis on CT scan, effectively mitigated the patient's risk of developing mediastinitis. Consequently, the patient received ongoing antibiotic therapy before and after surgery to forestall infectious complications. Specifically, the patient was administered cefixime 200 mg IV twice daily for 10 days.

The patient was discharged from our hospital on February 14, 2023, in good condition. As of the writing of this medical history, which transpired twelve months post-discharge, the patient's condition remained satisfactory, and he continued to exhibit normal growth and development appropriate for his age.

Applying our treatment method to similar cases has certain limitations. The European Society of Gastrointestinal Endoscopy (ESGE) advises conducting therapeutic esophagogastroduodenoscopy within 2 h, but no later than 6 h, in instances of foreign bodies causing complete esophageal obstruction, and within 24 h for cases of partial obstruction. Additionally, the ESGE recommends performing a computed tomography (CT) scan for all patients suspected of perforation or experiencing other complications. In comparison to x-ray imaging, CT scans offer advantages, as they can reveal larger volumes of soft tissue and fluid, which may otherwise obscure the limited calcium content found in fish bones (23).

In our case, after 12 days of admission to the hospital the foreign body was removed by endoscopic method.

The risk of developing complications was mitigated through the prompt initiation of antibiotic therapy and the placement of an endovascular prosthesis in the thoracic aorta.

Nine months later, a contrast-enhanced CT scan of the chest organs was conducted. The aorta exhibited uniform and complete opacification with contrast media. A stent graft was identified in the descending aorta, positioned at the level of the Th3-Th7 vertebral bodies. No discernible signs of aneurysm were observed.

## Data Availability

The original contributions presented in the study are included in the article/Supplementary Material, further inquiries can be directed to the corresponding authors.
